# *Drosophila tan* Encodes a Novel Hydrolase Required in Pigmentation and Vision

**DOI:** 10.1371/journal.pgen.0010063

**Published:** 2005-11-18

**Authors:** John R True, Shu-Dan Yeh, Bernhard T Hovemann, Tobias Kemme, Ian A Meinertzhagen, Tara N Edwards, Shian-Ren Liou, Qian Han, Jianyong Li

**Affiliations:** 1 Department of Ecology and Evolution, State University of New York, Stony Brook, New York, United States of America; 2 Fakultät für Chemie, Ruhr-Universität Bochum, Bochum, Germany; 3 Life Sciences Centre, Dalhousie University, Halifax, Nova Scotia, Canada; 4 Department of Biology, Dalhousie University, Halifax, Nova Scotia, Canada; 5 Department of Veterinary Pathology, College of Veterinary Medicine, University of Illinois, Urbana, Illinois, United States of America; Stanford University School of Medicine, United States of America

## Abstract

Many proteins are used repeatedly in development, but usually the function of the protein is similar in the different contexts. Here we report that the classical *Drosophila melanogaster* locus *tan* encodes a novel enzyme required for two very different cellular functions: hydrolysis of N-β-alanyl dopamine (NBAD) to dopamine during cuticular melanization, and hydrolysis of carcinine to histamine in the metabolism of photoreceptor neurotransmitter. We characterized two *tan*-like *P*-element insertions that failed to complement classical *tan* mutations. Both are inserted in the 5′ untranslated region of the previously uncharacterized gene *CG12120,* a putative homolog of fungal isopenicillin-N N-acyltransferase (EC 2.3.1.164). Both *P* insertions showed abnormally low transcription of the *CG12120* mRNA. Ectopic *CG12120* expression rescued *tan* mutant pigmentation phenotypes and caused the production of striking black melanin patterns. Electroretinogram and head histamine assays indicated that *CG12120* is required for hydrolysis of carcinine to histamine, which is required for histaminergic neurotransmission. Recombinant *CG12120* protein efficiently hydrolyzed both NBAD to dopamine and carcinine to histamine. We conclude that *D. melanogaster CG12120* corresponds to *tan*. This is, to our knowledge, the first molecular genetic characterization of NBAD hydrolase and carcinine hydrolase activity in any organism and is central to the understanding of pigmentation and photoreceptor function.

## Synopsis

True et al. describe the identification and characterization of the *Drosophila melanogaster* enzyme Tan. The gene encoding Tan was originally discovered in the early 20th century as a mutant strain lacking the dark pigmentation of wild-type flies, hence the name *tan*. Flies lacking Tan function also exhibited mysterious abnormalities in vision, for example, in responses to light. The new findings by True et al. help to explain the vastly different functions of Tan in pigmentation and vision. In the developing epidermal cells that secrete the adult cuticle, the enzyme encoded by *tan* is required for the production of dopamine, which is needed for dark melanin pigmentation. In the eye, the Tan enzyme converts carcinine, a modified form of the neurotransmitter histamine, back to histamine, which is necessary for the rapid and constant neurotransmission events involved in vision. These two enzyme activities have not been previously characterized in any organism. Surprisingly, Tan appears to be closely related to an enzyme in fungi that is used for production of the antibiotic penicillin.

## Introduction

One of the most important generalizations to emerge from contemporary developmental genetics is that gene products are typically used more than once during development. Usually multiple functions involve the protein performing the same task, e.g., activating transcription or binding to the cytoskeleton. A more surprising form of multifunctionality occurs in enzymes adapted to perform a catalytic function on different substrates in distinct developmental or metabolic pathways. The products encoded by the *Drosophila melanogaster ebony* and *tan* genes, mutations in which cause reciprocal pigmentation defects but related neurological phenotypes, have been proposed as such a system [1–6]. *ebony* encodes N-β-alanyl dopamine (NBAD) synthase, which in epidermal cells converts the melanin precursor dopamine to N-β-alanyl dopamine, a precursor to tan-colored pigment, during cuticle development in late pupae [2,6].

The molecular nature of the *tan* gene, however, has remained a mystery. The *tan* gene product has been proposed to catalyze the conversion of NBAD to dopamine via hydrolysis, which is the reverse of the activity catalyzed by the Ebony protein [2]. The reciprocal pigmentation effects of *tan* and *ebony* mutants can thus be explained: excess NBAD and deficient dopamine result in the abnormally light phenotype of *tan,* whereas excess dopamine and deficient NBAD result in the melanic phenotype of *ebony,* in which excess dopamine is converted to melanin.

The Ebony protein is related to a family of fungal and bacterial non-ribosomal peptide synthetases sharing ATP-dependent carboxy acid activation via acyladenylate and a thioester module that binds a 4′ phosphopantetheinyl cofactor during conversion from the apo to holo form [2,4]. Richardt et al. [4] demonstrated that Ebony protein is capable of capturing a number of biogenic amines, including histamine, and of binding these amines to β-alanine, as predicted from the biochemical genetic data. The extended lineage of *ebony* suggests that the putative *ebony–tan* circuit may be quite ancient. However, the apparent absence of NBAD in vertebrates suggests that the complementary functions of *ebony* and *tan* in dopamine metabolism and pigmentation may have been derived in protostomes or, more restrictively, in arthropods. More definitive evidence on the evolutionary history of this circuit could be provided by characterizing the *tan* locus. A further motivation to characterize the role of *tan* in pigment formation is provided by its potential involvement in pigment pattern evolution in insects [7,8].

Recent studies have begun to shed light on the neural functions of *tan* and *ebony*. The detailed characterization of Ebony function and expression [4,5] strongly suggests that the Ebony–Tan circuit plays a role in the metabolism of histamine, a photoreceptor neurotransmitter. The optomotor and visual defects of *ebony* mutants are consistent with reduced transmission from the terminals of the photoreceptors R1–R6. This possibility was initially suggested by the loss in *ebony* mutants of the “on” and “off” transients of electroretinograms (ERGs), first reported by Hotta and Benzer [9], but without explanation at that time. This defect is now attributable to the loss of transmission to lamina interneurons [10]. More recently, Richardt et al. [5] demonstrated that Ebony protein is expressed in non-neuronal cells of the first and second optic neuropiles, the lamina and medulla, respectively, in particular the epithelial glia of the lamina and the neuropile glia of the distal medulla. These glia are located at the sites of histamine release from the photoreceptors [5]. Collectively, the localization data and the biochemical function of Ebony as a general β-alanyl biogenic amine synthase [4] suggested that Ebony's role is to deactivate histamine by conjugating it with β-alanine, to form N-β-alanyl histamine, also known as carcinine, after histamine release from the photoreceptor terminal. Rapid histamine deactivation and reuptake are necessary for photoreceptor function because histamine release rates are high [11,12], whereas histamine synthesis and degradation are relatively slow [13].

Evidence for a role of the Ebony–Tan circuit in regulating histamine conjugation and regeneration in vivo prior to its presumed reuse by photoreceptors was provided by Borycz et al. [3], who demonstrated that fly heads rapidly convert microinjected histamine to carcinine and that *tan* mutants have abnormally high quantities of carcinine, hydrolysis of which is blocked. The same study also demonstrated that both *ebony* and *tan* mutants have substantially reduced head histamine content, as well as fewer synaptic vesicles at photoreceptor terminals. Together with the evidence on Ebony localization and function [4,5], these observations have led to a model of Ebony–Tan function in the fly's visual system. Histamine released at the photoreceptor terminals is captured by conversion to carcinine by Ebony in the surrounding glial cells. This carcinine is subsequently hydrolyzed by the product of the *tan* gene, which liberates the histamine for reuse by the presynaptic photoreceptor neuron. Neither the function nor the localization of Tan has yet been established, however.

Validation of this model requires molecular identification and characterization of the *tan* locus and its product, a putative NBAD/carcinine hydrolase that has yet to be identified in any organism. Here we present genetic and biochemical evidence that the *D. melanogaster tan* locus corresponds to predicted gene *CG12120* and confirm that it encodes a bifunctional NBAD/carcinine hydrolase. Tan/CG12120 is related to fungal isopenicillin-N N-acyltransferases (IATs), suggesting that like Ebony, insect NBAD/carcinine hydrolase also represents an ancient enzyme. We discuss the cellular and evolutionary implications of this discovery for models of both photoreceptor function and pigmentation development.

## Results

### Two *P*-element Insertions in *CG12120* Exhibit *tan*-Like Phenotypes and Fail to Complement *tan* Mutants

**Figure 1:**
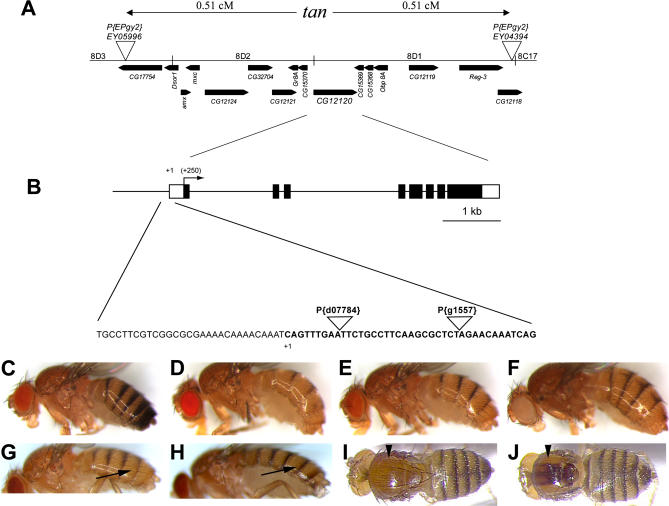
*P*-Element Insertions in *CG12120* Cause *tan*-Like Pigmentation Phenotypes and Ectopic Expression of *CG12120* Rescues the *tan* Phenotype and Causes Ectopic Melanin Formation (A) The *tan* region of the *D. melanogaster* X chromosome. Known and predicted genes with directions of transcription are depicted below the line. Cytological divisions and flanking *P*-element insertions, as well as meiotic mapping data (see text), are indicated above the line. (B) Transcription unit of predicted gene *CG12120*. Boxes indicate exons. Black indicates coding region. Transcription start site, inferred from EST data [15], is at the position designated +1. Start codon is at position +250. Below are indicated positions of *P{d07784}* and *P{g1557}* insertions, just 3′ to the transcription initiation site (+1) of *CG12120*. Transcription unit is indicated in bold. (C–F) Adult (3–5 d) female body coloration of (C) *Canton^S^* (wild-type), (D) *tan^5^,* (E) *w P{d07784}*, and (F) *w P{g1557}*. (G and H) *w tan^1^/w tan^1^; CyO/+; P{w^+^ C765-GAL4}/+* control female (G) and *w tan^1^/w tan^1^; P{w^+^ UAS-CG12120}/+; P{ w^+^ C765-GAL4}/+* female (H) showing rescue of abdomen phenotype (arrows). (I and J) *w/w; CyO/+; P{w^+^ pnr-GAL4}/+* control female (I) and *w/w; P{w^+^ UAS-CG12120}/+; P{w^+^ pnr-GAL4}/+* female (J) showing ectopic melanin pattern along dorsal midline (arrowheads). All flies depicted were 3–5 d of age.

The *tan* locus was originally mapped to the cytological interval 8C3-9E3 by the inclusion of *tan* in *Df(1)t282–1* [14]. We used meiotic mapping of local *P*-element insertions to determine that *tan* maps 0.51 cM proximal to *P*-element *P{EPgy2}EY04394* (near *CG12118*) at position 8D1 and 0.51 cM distal to *P*-element *P{EPgy2}EY05996* (near *CG17754*) at position 8D3 (Figure 1). This map interval contains 16 known or predicted genes. We subsequently obtained two new *P* insertions in this interval, *P{d07784}* [15] and *P{g1557}* [16], both of which were associated with a *tan*-like pigmentation phenotype (Figure 1E and 1F; compare with wild-type, Figure 1C, and *tan* mutant *tan^5^*, Figure 1D). One of these *P* inserts, *P{d07784}* was crossed with four classical *tan* mutants (*tan^1^*, *tan^2^*, *tan^4^*, and *tan^5^*) and failed to complement them for the pigmentation phenotype. These two *P* insertion lines were sequenced to determine the positions of the *P* insertions. In both lines, the *P*-element is inserted in the 5′ end of the first exon of predicted gene *CG12120,* at positions +9 and +21 bp, respectively (Figure 1B).

### *CG12120* Expression Rescues the *tan* Pigmentation Phenotype

The complete 2,009-bp *CG12120* cDNA was cloned into the *pUAST* vector [17] and used to transform a *yellow white (yw)* strain of *D. melanogaster*. Transformant lines were crossed into *y^+^w tan^+^* and *y^+^w tan* backgrounds. We tested whether *CG12120* expression under the control of the ubiquitous driver *C765-GAL4* [18] in a *tan^1^* background could rescue the pigmentation phenotype. *C765-GAL4*-mediated *CG12120* expression is sufficient to rescue the *tan^1^* pigmentation phenotype in the adult epidermis (Figure 1G and 1H). Similar rescue was seen with *C765-GAL4* driving *UAS-CG12120* in a *tan^5^* background, as well as with *patched-GAL4; UAS-CG12120* in both *tan^1^* and *tan^5^* backgrounds (data not shown).

### Ectopic *CG12120* Expression Produces Ectopic Melanin Pigmentation

We predicted that ectopic *tan* expression should result in ectopic melanin patterns by reversing the melanin-inhibiting role of *ebony* and thus providing dopamine for melanin synthesis [6–8]. To test this prediction, we drove *UAS-CG12120* expression with a panel of *GAL4* lines (complete dataset available upon request). A typical result is shown in Figure 1I and 1J. *pannier-GAL4; UAS-CG12120* caused a dark stripe of ectopic melanin to appear in the *pannier* pattern along the dorsal midline, especially evident in the notum (arrowheads in Figure 1I an 1J). This phenotype is similar to driving ectopic *yellow* expression in an *ebony* mutant background [6].

### *CG12120* Is Expressed in the Optic Lobes of Adult Flies

**Figure 2:**
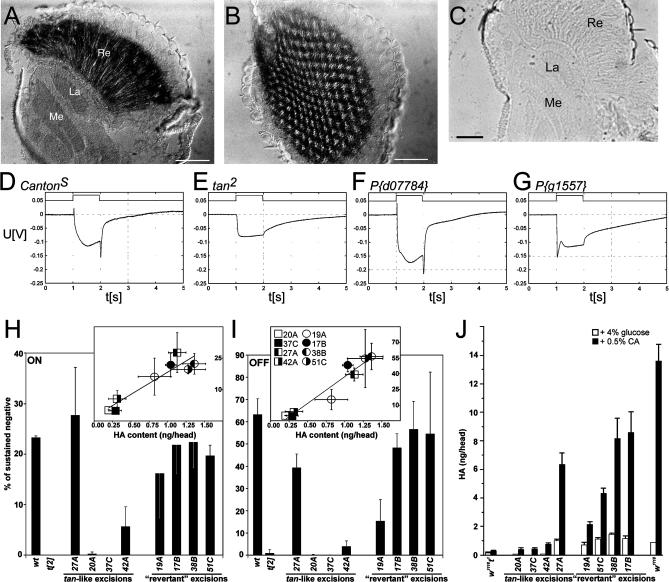
Tan Expression and Function in the Eye and Optic Lobes (A) RNA in situ hybridization with an antisense *tan* cDNA probe to wild-type adult head sections. Horizontal section cut through the eye and the optic lobes. (B) Sagittal section through the ommatidial array of the eye. (C) Control sense probe does not show significant staining (horizontal section). (D–G) ERG phenotypes of wild-type flies (D) and *tan* mutants (E–G). (D) *Canton^S^* (wild-type) ERG showing normal “on” and “off” transients. Upper trace in each recording indicates duration of light input stimulus (see Materials and Methods). (E) *tan^2^* mutant showing complete absence of “on” and “off” transients. (F) *P{d07784}* exhibits a normal “on” and “off” transient even though it has a *tan*-like pigmentation phenotype. (G) *P{g1557}* lacks “on” and “off” transients. (H and I) Normalized “on” (H) and “off” (I) transients for wild-type, *tan^2^ (t[2])*, *tan*-like *P* excision lines, and “revertant” *P* excision lines (see text) expressed as a percentage of the respective sustained negative potential. Insets: Correlation between head histamine content and the normalized magnitude of ERG ‘on’ and ‘off’ transients (see text). Error bars: ± 1 standard error in both dimensions. (J) Head histamine contents for *tan*-like and revertant *P* excision lines (see text), relative to control *w^1118^* and *tan^1^w^1118^* flies. Head histamine increased in all flies that drank 0.5% carcinine in 4% glucose (black bars), relative to controls that drank only 4% glucose (open bars). Revertant and *tan*-like excision lines are each arranged in rank order. Note that excision line flies that drank only 4% glucose have histamine contents too small to show above the abscissa. Error bars represent ± 1 standard error. La, lamina; Me, medulla; Re, retina. Scale bars = 50 μm.

In order to verify that *tan/CG12120* is expressed in the *Drosophila* visual system we performed in situ hybridization experiments using a digoxigenin-labeled *CG12120* cDNA antisense RNA probe (Figure 2). Head cryosections clearly revealed labeling in the retina. In horizontal sections the label appeared in thread-like structures resembling photoreceptor cell expression (Figure 2A). In sections that cut the retina in a sagittal plane, label showed the typical ommatidial structure of the retina (Figure 2B). Light areas resembling the ommatidial cavity were surrounded by dark label constituting a ring-like structure composed of photoreceptor cell somata. This pattern clearly indicates that the photoreceptor cell expression of *tan* differs from, and is complementary to, the lamina epithelial glial expression of *ebony* (see [5]).

### *tan* Mutants Exhibit Reduced ERG Transients

Like *ebony* mutants, *tan* flies lack normal “on” and “off” transients in their ERG ([9]; Figure 2E and 2G). In order to more thoroughly examine the association between *tan* pigmentation and ERG phenotypes, ERGs were recorded and the lamina “on” and “off” transients measured for the two *tan P* insertion lines, as well as for four *tan*-like and four “revertant” excisions of the *P{d07784}* insertion (see Materials and Methods). *P{d07784}* (Figure 2F) exhibited normal “on” and “off” ERG transients (compare with *Canton^S^*; Figure 2D). However *P{g1557}* (Figure 2G) lacked both “on” and “off” transients, somewhat similar to *tan^2^* (Figure 2E). For three of four *tan*-like *P* excision lines, normalized “on” transients (Figure 2H) reached values of only 0%–5.6%, compared with 23.3% for wild-type eyes, from which they differed significantly (*p* ≤ 0.001, *t*-test). ERG “off” transients for these same three lines were between 0.2% and 3.9%, compared with a wild-type value of 63.3%, also significantly different (*p* < 0.001). Excision line *27A* exhibited normal “on” transients, and markedly reduced, but not absent, “off” transients (Figure 2I). The amplitudes of these transients reached 27.7% (*p* >> 0.05) and 39.3% (*p* < 0.05), respectively, thus revealing partial rescue of the wild-type phenotype.

### ERG Transients Are Restored in Revertant *P* Excision Lines

Flies from three of four revertant *P* excision lines exhibited normalized transients comparable in amplitude with wild-type (Figure 2H and 2I), line *017B* reaching 21.8% (*p* >> 0.05), line *038B* 22.3% (*p* >> 0.05), and line *051C* 19.7% (*p* = 0.034) for “on,” compared with a wild-type *Canton^S^* value of 23.3% (Figure 2H). The normalized amplitudes of the “off” transients reached 48.3% in *017B* (*p* = 0.032), while in *038B* and *051C* the values were 56.6% and 54.5%, respectively (both *p* >> 0.05), compared with a wild-type transient value of 63.3% (Figure 2I). Line *019A* showed a less pronounced rescue effect: the “on” transient was restored, with a value of 16.2% (*p* > 0.05) (Figure 2H), while the “off” transient value of 15.3% remained significantly less than the wild-type level (*p* < 0.05) (Figure 2I).

### *tan*-Like *P* Excision Lines Have Reduced Head Histamine and Impaired Conversion of Carcinine to Histamine

Flies from the four *tan*-like *P* excision lines (*20A, 37C, 42A,* and *27A*) were given either 4% glucose to drink or 4% glucose laced with 0.5% carcinine. After drinking 4% glucose, all lines except *27A* had reduced head histamine contents compared with corresponding *w^1118^* control flies (Figure 2J). The reductions were to between 0.7% *(37C)* and 7.3% *(20A)* of *w^1118^* histamine levels. Line *27A* had, by contrast, 13% more head histamine than *w^1118^*. After the flies drank glucose plus carcinine, the head histamine contents were much larger than in flies that drank only glucose. These differences were significant for all excision lines (*p* < 0.05, *t*-test). The increases were not in proportion to the original head histamine content. The accumulated histamine levels after carcinine feeding in all *P* excision lines were far less than for the corresponding control *w^1118^* flies, in which the differences were 2.15 times greater than in the *tan*-like excision line *27A*. The differences between all excision alleles and *w^1118^* were significant (*p* < 0.001, *t*-test) in carcinine-fed flies. However, there was no significant difference in head histamine contents between the excision lines *20A, 37C,* and *42A* and flies from a control *w^1118^* *tan^1^* double-mutant line that also drank a 0.5 % carcinine solution (*p* > 0.005). After ingesting carcinine, *w^1118^* flies had a histamine head content 47 times greater than the *w^1118^* *tan^1^* controls, confirming the defective ability of flies mutant for *tan* to liberate histamine from exogenous carcinine.

### Revertant *P* Excision Lines Exhibit Partially Restored Head Histamine Levels and Conversion of Carcinine to Histamine

Flies from the four revertant *P* excision lines *(019A, 051C, 038B,* and *017B)* were also fed carcinine and control solutions. Control head histamine contents in flies that drank a 4.0% glucose solution were either similar to (*019A* and *051C: p* > 0.05, *t*-test) or even slightly greater than (*017B* and *038B: p* < 0.005) those in *w^1118^* control flies (Figure 2J). After drinking carcinine, the head histamine increased in all revertant lines. The increases were significant compared with controls that did not drink carcinine (*t*-test, *p* < 0.005). Compared with *w^1118^* control flies that also drank carcinine, the increases in head histamine were less, by between 16% *(019A)* and 63% *(017B)* of *w^1118^* levels. The rank order in head histamine increases was roughly the same as the rank-ordered original head histamine contents in control flies before drinking carcinine. Thus, fly lines in which *tan* function was rescued most completely with respect to control head histamine content also had the largest histamine increases after drinking carcinine.

### Correlation of Total Histamine Content and Normalized ERG Transients

Values of normalized transients were plotted against the total amount of histamine per head for the corresponding *tan*-like and revertant excision lines (insets, Figure 2H, I). The relationship between the size of the transients and the histamine content was approximately linear for both line types, supported by high regression coefficients (0.87 and 0.93 for *tan*-like and revertant excisions, respectively). Thus, in a general way, the more head histamine made available by *tan* function, the larger the ERG transients generated by the release of histamine during transmission in the lamina. The correlation coefficients for both relationships were greater than 0.87. The “off” transients were more sensitive to head histamine than were the “on” transients. Values fit a linear relationships having the equations *y* = 21.31*x*_ON_ − 2.96 (*R* ^2^ = 0.87) for the “on” transients and *y* = 51.13*x*_OFF_ − 13.92 (*R* ^2^ = 0.93) for the “off” transients, where *y* represents histamine content and *x*_ON_ and *x*_OFF_ represent the normalized “on” and “off” transients, respectively. This difference supports the separable origins of the transients [19]. The relationship takes no account of the histamine located outside the visual system, of which there are several sources [20], which may explain the residual histamine content (0.2–0.3 ng/head) when the transients were zero.

### The CG12120 Protein Is Conserved among Insects and Is Related to Fungal IAT

**Figure 3:**
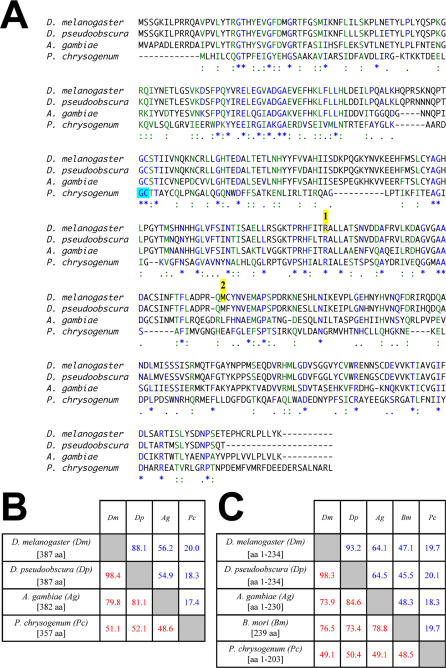
*Drosophila CG12120* Is Conserved across Insects and Is Related to Fungal IAT (A) Alignment of *D. melanogaster, D. pseudoobscura,* and *A. gambiae* CG12120 orthologs and *P. chrysogenum* IAT protein. Residues in blue are identical (*) across all four species, and residues in green are functionally similar, showing strong (:) or weak (.) similarity. Residue highlighted “1” indicates conserved arginine residue at position 217 that is mutated to proline in *tan^1^* and *tan^4^* mutants. Residue highlighted “2” indicates methionine residue at position 256 that is mutated to isoleucine in *tan^5^* mutant. Cyan rectangle indicates auto-processing site of *P. chrysogenum* at which pro-IAT is cleaved between glycine 102 and cysteine 103. (B) Percent sequence identity (blue in upper right) and sequence similarity (red in lower left) among *Drosophila* spp., *A. gambiae,* and *P. chrysogenum* proteins. (C) Percent sequence identity (blue in upper right) and sequence similarity (red in lower left) of N-terminal *Drosophila* spp., *A. gambiae,* and *P. chrysogenum* proteins with presumptive *B. mori* CG12120 ortholog (see text).

*CG12120* encodes a 387-amino-acid polypeptide with a predicted molecular weight of approximately 44 kD. *CG12120* homologs are present in all sequenced *Drosophila* genomes, as well as in the genomes of *Anopheles gambiae* and *Bombyx mori*. Sequence identity among insect CG12120 proteins extends over the entire length of the protein for the two Diptera (79.8% identical between *D. melanogaster* and *A. gambiae;* Figure 3A and 3B) and the first 230–240 amino acids between dipteran and *Bombyx mori* sequences (45%–48% identical between dipterans and *B. mori;* Figure 3C). The *B. mori* sequence may be an incomplete fragment, to be clarified pending the release of an annotated version of the genome sequence. Interestingly, several fungal IATs (*Pencillium chrysogenum* shown in Figure 3A and 3B) are approximately 50% similar (based on Gonnet series in CLUSTALW [21]) and 20% identical to insect CG12120 proteins, suggesting the conservation of a very ancient gene present in the common ancestor of fungi and metazoans. Fungal IATs are one of three enzymes in the penicillin biosynthetic pathway and catalyze the substitution of the L-α-aminoadipyl side chain of isopenicillin-N with aromatic acyl side chains [22]. A BLAST search of *P. chrysogenum* IAT to the *D. melanogaster* genome turned up two other proteins, but these have less substantial similarity to IAT: CG12140, a predicted electron-transferring flavoprotein dehydrogenase (25% identical, 38% similar over a 148-amino-acid region from residues 90–228 of IAT), and CG8864, a predicted monooxygenase/oxidoreductase electron transporter (29% identical, 38% similar over a 78-amino-acid region from residues 224–289 of IAT). Therefore, it appears that CG12120 is the only protein in the *Drosophila* genome with strong homology to fungal IATs.

The presence of completely conserved sites between metazoans and fungi through virtually the entire length of the CG12120/IAT proteins strongly suggests that the molecular mechanism underlying enzyme activity has been conserved, even though these proteins function in very different pathways in fungi and metazoans and in two different functional pathways even within insects. One site of particular interest is the conserved glycine–cysteine domain at position 102–103, which is the site of autocatalytic processing of fungal IATs [23]. Conservation of this domain among insects suggests that autocatalytic cleavage may also be present in CG12120**.**

The CG12120 amino acid sequence was highly conserved between wild-type flies and the five classical *tan* mutants that were sequenced. Only two amino acid substitutions were found. In *tan^1^* and *tan^4^,* a highly conserved arginine is changed to proline at position 217 (Figure 3A). Since these two mutants did not exhibit extremely different levels of *CG12120* transcript from wild-type (data not shown), it is likely that this substitution of an evolutionarily conserved amino acid is functionally important and responsible for the *tan* phenotype. One other substitution, a methionine to isoleucine substitution at position 256 in *tan^5^,* a region of little sequence conservation except among *Drosophila* species, is not predicted to cause an extreme functional change in the protein. The *tan^5^* allele may represent a more profound disruption of the *CG12120* locus, given that PCR amplification of the 3′ end of this allele, including the last two exons, was not successful in several attempts.

### CG12120 Possesses Both NBAD Hydrolase and N-β-Alanyl Histamine Hydrolase Activity

**Figure 4:**
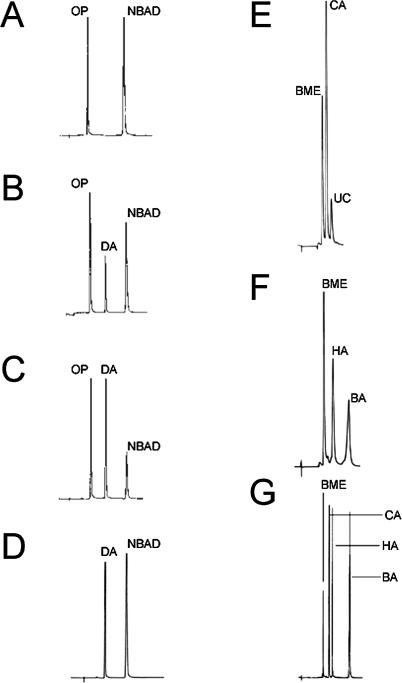
The *CG12120* Gene Product Possesses NBAD Hydrolase and N-β-Alanyl Histamine (Carcinine) Hydrolase Activities HPLC-ED chromatograms from activity assays. (A) Lysate from control (recombinant AGT) transfected *Sf9* cells exhibited no activity. Oxidation product (OP) peak denotes uncharacterized presumptive oxidation product of NBAD present in NBAD substrate in all experiments. (B) NBAD incubated with CG12120 protein purified from *Sf9* cells expressing *CG12120* baculovirus construct after 20 min incubation with NBAD. Dopamine (DA) peak is evident. (C) Same experiment as in (B) but after 50 min incubation, showing increased accumulation of dopamine and depletion of NBAD. (D) Control HPLC chromatogram containing dopamine and NBAD standards. (E) Extract from control (recombinant AGT) transfected *Sf9* cells exhibits no production of β-alanine and histamine when incubated with carcinine (CA). Unknown contaminant (UC) peak denotes an unknown contaminant in carcinine. β-mercaptoethanol (BME) peak denotes β-mercaptoethanol present in the reaction solution. (F) Extract from *Sf9* cells transfected with *CG12120* baculovirus incubated with carcinine demonstrates production of histamine (HA) and β-alanine (BA). Under the applied conditions, essentially all carcinine was hydrolyzed to histamine and β-alanine. (G) Control HPLC chromatogram containing β-mercaptoethanol, carcinine, histamine, and β-alanine standards. β-alanine and histamine detection were enabled by OPT conjugation (see Materials and Methods).

The *tan* gene product is predicted to encode a multifunctional hydrolase that catalyzes the hydrolysis of NBAD into β-alanine and dopamine, and the hydrolysis of carcinine (N-β-alanyl histamine) into β-alanine and histamine, respectively. To test whether CG12120 possesses these predicted activities, we produced recombinant CG12120 protein using a baculovirus expression system in insect cell culture (Figure 4). After soluble proteins from either uninfected *Spodoptera frugiperda (Sf9)* insect cells or *Sf9* cells infected with an alanine glyoxylate transaminase (AGT) recombinant baculovirus were mixed into a NBAD solution. Production of dopamine in the reaction mixture was not observed (Figure 4A), suggesting that *Sf9* cells do not have a protein capable of mediating NBAD hydrolysis, and infection of baculovirus itself also did not stimulate the production of a protein with NBAD-hydrolyzing activity (data not shown). In contrast, when soluble proteins from *CG12120* recombinant baculovirus-infected cells were mixed into a NBAD solution, accumulation of dopamine in the reaction mixture was observed and the amounts of dopamine produced in the reaction mixture were approximately proportional to the applied incubation periods (Figure 4B and 4C). During hydrolysis, an equal amount of β-alanine was produced in the reaction mixture (data not shown), but could not be detected electrochemically, because β-alanine is not electrochemically active.

A similar assay was used to examine carcinine hydrolysis. In this case, detection of β-alanine and histamine products was enabled by o-phthaldialdehyde thiol (OPT) conjugation (see Materials and Methods). After proteins from uninfected (not shown) or AGT recombinant baculovirus-infected insect cells were mixed into a solution of carcinine, hydrolysis of the carcinine was not observed (Figure 4E). However, after soluble proteins from *CG12120* recombinant baculovirus-infected cells were mixed into a carcinine solution, rapid accumulation of β-alanine and histamine was indeed observed in the reaction mixture (Figure 4F). Hydrolysis of both NBAD and carcinine by soluble proteins from *CG12120* recombinant baculovirus-infected insect cells provides direct and convincing evidence for the NBAD and carcinine hydrolase identity of the CG12120 protein.

## Discussion

The molecular identity of *tan* has been a longstanding question critical to the biology of melanin pigmentation and synaptic transmission at photoreceptors in insects. We have provided genetic, developmental, and biochemical evidence that the predicted *D. melanogaster* gene *CG12120* encodes *tan*. *P* insertions in the first exon of *CG12120* are associated with *tan*-like phenotypes and fail to complement classical *tan* mutants. Reduction of *CG12120* transcript levels is correlated with *tan* pigmentation, ERG, carcinine hydrolysis, and histamine phenotypes. Ectopic expression of *CG12120* in transgenic *D. melanogaster* rescues the *tan* pigmentation phenotype in *tan* mutant backgrounds and causes ectopic melanization, analogous to loss of *ebony* function, in wild-type backgrounds. The CG12120 protein exhibits the two predicted enzyme activities, NBAD hydrolase and carcinine hydrolase, in vitro. Taken together, our data demonstrate that *CG12120* is *tan*.

### The Tan–Ebony Circuit Occupies a Pivotal Position in Melanin Biosynthesis

**Figure 5:**
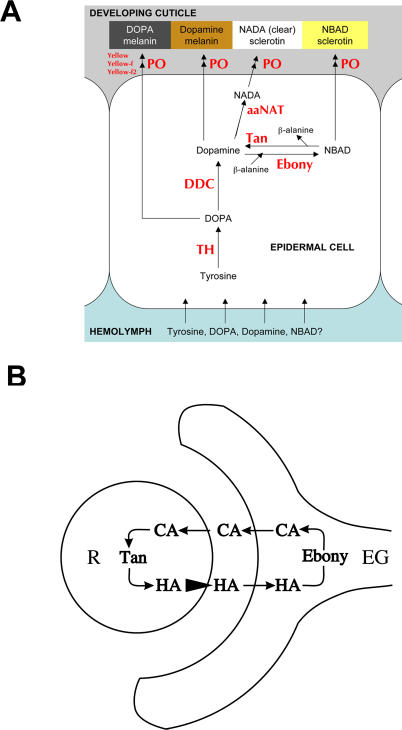
Tan Functions in Diverse Developmental and Metabolic Pathways (A) In developing adult epidermal cells, Tan catalyzes the production of dopamine from NBAD during pigment development. This is one of four parallel pathways by which dopa or dopamine derivatives are secreted into the developing cuticle as precursors for distinct pigments. aaNAT, arylalkylamine-N-acetyl transferase; DDC, dopa decarboxylase; NADA, N-acetyl dopamine; PO, phenol oxidase; TH, tyrosine hydroxylase. (B) In the photoreceptor, Tan catalyzes the hydrolysis of N-β-alanyl histamine (carcinine) to histamine for re-uptake by the presynaptic photoreceptor cell (R). CA, carcinine; EG, epithelial glial cell; HA, histamine.

The molecular identification of *tan* helps clarify a crucial step in dopamine metabolism and melanin biosynthesis in epidermal cells. All developing adult epidermal cells in insects are capable of secreting catecholamine precursors of melanin and sclerotin, and current models [1,6,24] propose that the patterns of adult melanin reflect the differential spatial regulation of four parallel branches from the core dopamine pathway catalyzed by tyrosine hydroxylase and dopa decarboxylase (Figure 5A). One of the four branches produces dopa melanin, which is under the control of *yellow* [6,25], the exact function of which is unknown, and at least two Yellow-related proteins, Yellow-f and Yellow-f2, which convert dopachrome to 5,6-dihydroxyindole [26]. Dopamine is also secreted and converted into dopamine melanin through an as yet uncharacterized pathway. Areas of the cuticle that are not melanized secrete NBAD, produced by the action of the Ebony protein [2,6], resulting in yellow or light tan cuticle, or N-acetyl dopamine, produced by the action of the arylalkylamine N-acyltransferases [27], which results in transparent cuticle (J. R. T., unpublished data). All of these precursors are extracellularly polymerized and crosslinked to cuticle proteins, probably through the action of a common set of enzymes, including phenol oxidases [28], the functions of which in the developing cuticle are not well characterized. Tyrosine and catecholamines are also provided to some degree from the hemolymph [1,29], and a hemolymph supply of melanin precursors is required for wing pigmentation [24].

Normal melanization depends in part on Tan function to provide dopamine by hydrolyzing sequestered NBAD. It is currently unclear why this dopamine is produced from NBAD rather than directly from dopa by dopa decarboxylase. One possible explanation for an Ebony–Tan “shunt” would be if epidermal cells require rapid or precise temporal regulation of dopamine secretion during cuticle development. For example, long-term sequestration of dopamine awaiting this developmental time window could be injurious to the cell. Alternatively, conversion of dopamine to NBAD by Ebony may be a constitutive ancestral state in insects, and conversion of some of this NBAD back to dopamine for melanin production may be a derived condition in some insects. NBAD synthase activity has been demonstrated in lepidopterans [30], in which NBAD is a precursor to yellow papiliochrome pigment. Isolation and functional characterization of *tan* and *ebony* gene homologs from more basal insects will be needed to test these alternative hypotheses.

The production of dopamine melanin depends on Tan function, which in turn depends on Ebony to produce its substrate. As predicted by this relationship, *ebony* is epistatic to *tan* (J. R. T., unpublished data). Production of melanin from both dopa and dopamine is an apparent degeneracy that occurs in insects but not vertebrates, which produce melanin primarily from L-dopa [31]. The final dark black color of many insects reflects contributions of both types of melanin, which continuously darken during cuticle maturation and hardening. There is evidence in *D. melanogaster* that the two melanin pathways are not independent. The presence of Ebony appears to determine whether melanin will be produced, even in the presence of ectopic Yellow, which gains access to the core dopamine pathway upstream at the dopa stage. Only in the absence of Ebony function is ectopic Yellow able to promote ectopic melanin production [6]. This suggests that normally most dopa is converted to dopamine and then to NBAD (or N-acetyl dopamine), but when the dopamine-to-NBAD step is blocked in an *ebony* mutant more dopa may be available for Yellow-mediated conversion to dopa melanin, possibly because of product inhibition of dopa decarboxylase [32]. Note that back-conversion of dopamine to dopa has not been observed in insects [1]. *ebony* mutants accumulate excess levels of dopamine, which is shunted to dopamine melanin. This mechanism has long been a candidate for naturally occurring melanism, which is an extremely common type of polymorphism in insects [7,33]. Thus, *ebony* itself is a candidate gene for such polymorphisms. However, *D. melanogaster ebony* mutants do not show the complete dominance typical of naturally occurring melanic alleles in other insects. Another important candidate is *tan,* which we have demonstrated here is mutable, via gain of function, to dominant production of ectopic melanin.

### Role of *tan* in Insect Vision

Compared with melanin biosynthesis, less can be said of *tan*'s involvement in histamine metabolism, but our findings do help to explain the action of *tan,* and thus clarify the Ebony–Tan pathway, in the visual system. The essential feature is that Tan localizes to the photoreceptor cells, and thus presumably their synaptic terminals, in a complementary position to that of Ebony, which localizes to the surrounding glial cells. This result helps explain early mosaic studies indicating that *tan* acts autonomously either within or very close to the eye [34]. Thus, histamine released from the photoreceptor terminals must apparently enter the epithelial glia and become converted to carcinine by Ebony, and the carcinine must return to the photoreceptor terminal, where hydrolysis can liberate histamine (Figure 5B). Uptake mechanisms and pathways are unknown, not only for histamine and carcinine, but also for the β-alanine co-liberated from carcinine by photoreceptor Tan, and identification of these now constitutes a next line of inquiry.

Histamine liberated in the photoreceptor terminal is presumed to finally become available for pumping into newly endocytosed synaptic vesicles. The site of the latter function is now clear: in the lamina, endocytotic recovery of new synaptic vesicles is localized to the stalk region of capitate projections [35], invaginations of the photoreceptor terminal, from epithelial glia [36]. Mutant *tan^1^* flies have significantly more penetrating capitate projections than wild-type flies [37], a phenotype that has been used to suggest that the capitate projection is an integrated recycling organelle site for the endocytotic retrieval of membrane and the recycling of histamine [35]. Mutant *tan* flies have been suggested to lack ERG transients because they have an insufficient pool of photoreceptor histamine to release [3], but the differential action we report in *tan* alleles for the “on” and “off” transients suggests that this may at best be a partial explanation.

### Tan Is the First NBAD/Carcinine Hydrolase Characterized in Any Organism

Like Ebony [2], Tan is related to a microbial protein, in this case an enzyme involved in penicillin synthesis. Given their close reciprocal functions in dopamine and histamine metabolism, it is tempting to speculate that *ebony* and *tan* are evolutionarily ancient molecular partners in insects, and possibly in arthropods or even ecdysozoans. If this is the case, then the two genes may have been co-opted together or consecutively from a microbial genome, perhaps at the base of the metazoan lineage. Alternatively, *tan* and *ebony* could have descended from genes present in the common ancestor of fungi and metazoans, in which case they have apparently been lost in the chordate or deuterostome lineage. There are no proteins related in sequence to Tan and Ebony in any vertebrate genome sequenced to date, and NBAD has not been found in vertebrates. Carcinine was originally characterized in the crab *Carcinus maenas* [38], but its biological function until recently has been a mystery. Carcinine possesses several pharmacological properties in mammals, including antioxidant effects [39] and cardiac vasodilation [40], but an endogenous role for this compound has not been demonstrated in any deuterostome.

Characterization of the structure and function of the Tan/CG12120 protein, including the conserved putative autoprocessing site at positions 102–103 and the conserved arginine residue that is mutated to a proline in *tan^1^* and *tan^4^* mutants, will help reveal any possible conservation of function of this protein in insects and fungi. As further arthropod and protostome genomes are sequenced, the presence or absence and sequence evolution of *tan/CG12120* homologs will help indicate whether other invertebrate species utilize this protein for melanin production and/or neurotransmitter metabolism. An intriguing possibility in insects is that coevolution of pigment patterns and behaviors [7] may have involved *tan* and other genes that have pleiotropic actions in both pigmentation and the nervous system.

### Conclusions

We provide genetic, developmental, neurophysiological, and biochemical evidence that *D. melanogaster tan* corresponds to the predicted gene *CG12120*. *tan* encodes a multifunctional enzyme that hydrolyzes both NBAD to dopamine and carcinine (N-β-alanyl histamine) to histamine. In this study, this enzyme is characterized at the molecular level for the first time, to our knowledge, in any organism. Confirmation of these two enzyme activities of the Tan protein provides important clarification of the pleiotropic function of this gene in pigment development and in histamine metabolism at the photoreceptor organ. *tan* is also an important candidate gene for melanin pattern polymorphisms and species differences. Further study of Tan function in photoreceptor neurons will help clarify how transmitter released by photoreceptors is recovered for reuse during insect vision.

## Materials and Methods

### *Drosophila* strains and culture.

All *D. melanogaster* crosses were performed at 25 °C (23 °C for histamine assays) with a 12 h light:12 h dark cycle. Flies were cultured on standard corn meal/molasses/agar medium.

*C765-GAL4* [18] was provided by S. Carroll (University of Wisconsin, Madison, Wisconsin, United States). *pannier-GAL4* [41] was provided by G. Morata (Universidad Autonoma de Madrid, Madrid, Spain). *Canton^S^, Oregon^R^, w^1118^; +; P{Δ2–3}, TM3, Sb/TM6B,* and *Pro,os/FM6,B,w* were provided by W. Eanes (State University of New York, Stony Brook, New York, United States). *w^1118^ P{Mae-UAS.6.11} CG12120[g1557](P{g1557})* [16] was provided by U. Schäfer (Max-Planck-Institut für Biophysikalische Chemie, Göttingen, Germany). The following lines were provided by the Bloomington *Drosophila* StockCenter (http://flystocks.bio.indiana.edu/): *w^1118^, P{w[+mC]=XP} CG12120[d07784] (P{d07784}), w^1118^, t ^1^, t ^2^ v ^1^ f ^1^ (t ^2^), t ^3^, br ^1^ w ^e^ ec ^1^ rb ^1^ t ^4^/FM1 lz ^4^ (t ^4^), t ^5^ v ^1^ r ^1^/FM7c (t ^5^), P{EPgy2}EY04394,* and *P{EPgy2}EY05996*.

All flies were examined at 3–5 d of age, after the full adult pigmentation appeared. *UAS-CG12120* rescue and ectopic expression genotypes showed very little variation. Rescue and ectopic expression phenotypes occurred in 100% of the flies that inherited both the *GAL4* and *UAS-CG12120* elements.

### *P* excision lines.

*w^1118^, P{XP}CG12120 ^d07784^* females were crossed to *w^1118^; +; P{Δ2–3}, TM3, Sb/TM6B* males, and dysgenic male F_1_s were crossed to *Pro,os/FM6,B,w* females. Female F_2_ progeny lacking eye color (w^1118^, P*/FM6 B w; +; +) were crossed to *FM6,B,w/ *Y males, and their non-*FM6* male progeny were inspected for pigmentation phenotype. Four *tan*-like lines and four “revertant” lines with wild-type pigmentation were chosen for further analysis.

The four excisions classified as *tan*-like all contained imprecise excisions, two of which, *20A* and *37C,* contained large deletions (953 bp and 1,641 bp, respectively) that included the presumptive promoter region. The other two *tan*-like excisions, *27A* and *42A,* left small insertions (38 bp and 77 bp, respectively) at the *P*-element site (data available upon request). All four revertant *P* excisions were also imprecise, leaving *P*-element fragments ranging from 12 bp to 1.2 kb in size (data available upon request), but none of these contained deletions of the endogenous *CG12120* transcription unit or promoter region.

The *tan*-like excision lines showed significantly lower *CG12120* mRNA levels on average than wild-type lines by quantitative reverse transcriptase PCR assay (data not shown). Revertant excision lines showed no differences on average from wild-type lines in mean *CG12120* mRNA expression levels. These results were found at two different developmental stages, 60–75 h after puparium formation and 0–8 h after eclosion. The complete *CG12120* mRNA expression dataset is available upon request.

### Plasmid constructs.

For *UAS-CG12120,* the complete *CG12120* cDNA was obtained from the *Drosophila* Genomics Resource Center (http://dgrc.cgb.indiana.edu/) as clone *RH41996* (barcode 17763), consisting of the 2,009-bp *CG12120* cDNA in plasmid vector *pFLC-1*. A 2,133-bp NotI-Acc65I fragment containing the *CG12120* cDNA was cloned into the *pUAST* vector [17] to produce the *UAS-CG12120* construct. This construct was used to transform a *D. melanogaster yw* host strain as described [42], and transformant lines were homozygosed, mapped, and crossed into a *y^+^w* background for *tan* rescue and ectopic *CG12120* expression experiments.

For the CG12120 baculovirus expression construct, a 1,568-bp XbaI-BstBI fragment, containing the complete 1,164-bp predicted *CG12120* ORF, was cloned from *RH41996* into the *pBlueBac 4.5* baculovirus expression vector (Invitrogen, Carlsbad, California, United States). Then, in order to place the start codon as close as possible to the pBlueBac polyhedrin promoter, the *CG12120* cDNA was amplified from the *pBlueBac 4.5–CG12120* clone using a forward primer (5′-GCT AGC **ATG** TCC TCC TTA AAG ATC CTG-3′) containing a NheI restriction site (underlined), and a reverse primer (5′-AAG CTT CTA CTT GTA GAG CAG CGG CAG-3′) containing a HindIII restriction site (underlined). The start codon of the *CG12120* ORF is indicated in bold in the forward primer. The amplified DNA fragment was inserted into a *PCR2.1-TOPO TA* cloning vector and then cloned into *pBlueBac 4.5* between the NheI and HindIII restriction sites. The recombinant transfer vectors were sequenced and verified to ensure that the inserted genes were in-frame and controlled under the downstream polyhedrin promoter. Recombinant *NP 572543 (CG12120) pBlueBac 4.5* transfer vector was cotransfected with linearized *Bac-N-Blue* (*AcMNPV*, *Autographa californica* multiple nuclear polyhedrosis virus) viral DNA to *Sf 9* insect cells (Invitrogen) to generate recombinant *CG12120* baculovirus. The recombinant baculovirus was purified by the plaque assay procedure.

### Quantitative RT-PCR.

RNA was isolated from 20–30 individuals sorted by sex from two stages: pooled P8–P11 stage pupae (roughly 60–75 h after puparium formation, when eye color is present but macrochaetes and body cuticle are not yet pigmented [43]), and adults 0–8 h after eclosion. RNA isolation used a Stratagene (La Jolla, California, United States) Absolutely RNA Microprep kit and protocol. RNA yields were quantified by OD_260_ reading on a spectrophotometer. For each RT-PCR reaction, 300 ng was loaded. One-step quantitative RT-PCR analysis used the SYBR-green-based reagents and protocols in the Stratagene One-Step Brilliant QRT-PCR kit. The following RT-PCR products were quantified: for *CG12120,* a 117-bp fragment from position 327 to 443 of the *CG12120* cDNA amplified using forward primer 5′-CTT CGA TAT GGG TCG CAC ATT TGG-3′ and reverse primer 5′-TTG TAG ATC TGC CTG CCT TTT GG-3′; for α*-GPDH (CG9042),* a 187-bp fragment from position 1210 to 1396 of the *GPDH-RC* cDNA [15] using forward primer 5′-ATC TGA TCA CGA CGT GTT ACG GTG-3′ and reverse primer 5′-AAC AGG GGG AAT TTG TCC TCC AGA C-3′; and for *RPII-215 (CG1554),* a 211-bp fragment from position 2674 to 2884 of the *RPII-215* cDNA using forward primer 5′-TAT CCC AGG TTA TTG CTT GTG TGG G-3′ and reverse primer 5′-GCA GTA TCG ATA AGA CCT TCA CGA CC-3′. Quantitative RT-PCR was performed and product yields were quantified on a Stratagene MX3000P real-time thermal cycler connected to a Gateway PC laptop computer running version 2.0 of the MX3000P software. The following amplification procedure was used: 50 °C for 20 min (RT step), 95 °C for 15 min, 40 amplification cycles (94 °C for 30 s, 53 °C for 30 s, and 72 °C for 30 s), and 79 °C for 11 s (fluorescence reading), followed by melting curve analysis to confirm expected product *T*_m_. Controls without reverse transcriptase were run to estimate background signal, if any, due to amplification from genomic DNA. These background amounts were generally three to four orders of magnitude below experimental reactions and were subtracted from yields of experimental reactions prior to the calculation of *CG12120/RPII-215* and *CG12120/GPDH* ratios.

### ERG recordings.

Flies were immobilized in cut-off pipette tips with the head protruding from the opening. The indifferent and recording electrodes, filled with *Drosophila* Ringer's solution, were inserted into the posterior part of the head capsule and placed on the surface of the retina, respectively. After a stable baseline was obtained, the light impulse was triggered by removing a shutter from a Schott KL 150 B light source with a Xenophot 150-W halogen photo optic lamp (Osram, Augsburg, Germany), thus directing the light beam onto the fly's eye. The stimulus lasted for 1 s, and this cycle was repeated ten times for each individual fly after allowing the signal to return to baseline. Potentials were recorded over a 5-s time frame by a Hameg Instruments (Mainhausen, Germany) digital oscilloscope and stored with Hameg SP107 software.

ERG plots for individual flies were obtained by calculating the mean of the ten light/dark cycles. To normalize the transients, the sustained negative potential (which reports the photoreceptor response that drives transmission in the lamina, represented by the transients [10,44]), was determined from the averaged potential 20 ms prior to the offset of the light stimulus, and the relative size of the transients was then calculated as a percentage of this sustained negative potential.

### In situ hybridization to RNA.

For in situ hybridization, fly heads were mounted in Tissue-Tek O.C.T. compound (Microm, Walldorf, Germany) and were shock-frozen in liquid nitrogen. Sections of 10 μm were cut and fixed with 4% paraformaldehyde. After acetylation and prehybridization, subsequent hybridization with a digoxigenin-labeled RNA *CG12120*-cDNA probe was performed overnight at 55 °C. Specimens were blocked with normal goat serum in Tris-buffer saline/0.1% Triton X-100 and were then treated with an alkaline phosphatase-coupled anti-digoxigenin antiserum (1:1,000 dilution in Tris-buffer saline/0.1% Triton X-100). NBT/BCIP color development was controlled under the microscope for 20–60 min.

### Histamine assays.

Flies for histamine determinations were aged for at least 3 d prior to preparation for high performance liquid chromatography (HPLC) to ensure the completion of the critical period for lamina development [45], when histamine content has stabilized (J. Borycz and I. A. M., unpublished data). To determine Tan function by carcinine conversion to histamine, flies were dehydrated for 2 h prior to feeding with aqueous solutions of 4% glucose or 0.5% carcinine in glucose, and then allowed to drink from these solutions overnight for 16 h. Flies were collected 2 h after lights on, then rapidly frozen and stored at −80 °C until assayed by HPLC. To compare head histamine with the reduction in lamina ERG transients, head histamine was determined from flies that were fed on medium only.

In all cases, histamine determinations were performed on groups of 20–50 isolated heads using HPLC with electrochemical detection as reported for *D. melanogaster* [3,13,46], and values are reported for the means of 3–12 such samples.

### Recombinant *CG12120* expression.

*Sf 9* cells were cultured in 25-cm culture flasks in the presence of 10 ml of TNM-FH medium containing 10% fetal bovine serum (Invitrogen). High titer recombinant *CG12120* baculovirus was inoculated into the cell culture at 70% confluence. *Sf 9* cells were harvested 3 d post-inoculation by centrifugation (800 *g* for 15 min at 4 °C). The pellet cells were washed twice with PBS. Cells were lysed by sonication in 50 mM phosphate buffer. The lysate was centrifuged at 20,000 *g* for 20 min to obtain supernatant that was subsequently used for NBAD and carcinine hydrolyase activity assays.

### Enzyme activity assays.

Cell lysate supernatant from recombinant *tan* baculovirus-infected cells was assayed for NBAD and carcinine hydrolase activities. Cell lysate supernatant from AGT baculovirus-infected cells served as a control. A typical reaction mixture consisted of 50 μl of cell lysate supernatant, 100 μl of phosphate buffer (50 mM [pH 7.0]), and 50 μl of 8 mM NBAD (provided by the National Institute of Mental Health Chemical Synthesis and Drug Supply Program; http://nimh-repository.rti.org/) or N-β-alanyl histamine (a kind gift of M. Feigel, Ruhr-Universität Bochum, Bochum, Germany), prepared in 50 mM phosphate buffer. At different incubation periods, 50 μl of reaction mixture was withdrawn and mixed with an equal volume of 0.8 M formic acid (NBAD reactions) or an equal volume of absolute ethanol (N-β-alanyl histamine reactions) to stop the enzymatic reaction. The formic-acid-treated reaction mixture was centrifuged at 20,000 *g* for 15 min at 4 °C, and supernatant was analyzed by HPLC with electrochemical detection. Hydrolase activity to NBAD was determined based on the detection of dopamine in the reaction mixture, and hydrolase activity to N-β-alanyl histamine was determined based on the detection of OPT β-alanine and OPT histamine conjugates [47].

## Supporting Information

### Accession Numbers

The GenBank (http://www.ncbi.nlm.nih.gov/Genbank/) accession numbers for the gene products discussed in this paper are *A. gambiae* CG12120 (AAAB01008846), *B. mori* CG12120 (BAAB070172112), *D. melanogaster* CG12120 (NM 132315), and *D. pseudoobscura* CG12120 (CH379064). The Swiss-Prot (http://www.ebi.ac.uk/swissprot/) accession number for *P. chrysogenum* IAT is P15802.

## Abbreviations

AGT: alanine glyoxylate transaminase
ERG: electroretinogram
HPLC: high performance liquid chromatography
IAT: isopenicillin-N N-acyltransferase
NBAD: N-β-alanyl dopamine
OPT: o-phthaldialdehyde thiol
*Sf9*: *Spodoptera frugiperda*
*yw*: *yellow white*
